# The Coexistence of Oculocutaneous Albinism with Schizophrenia

**DOI:** 10.7759/cureus.6617

**Published:** 2020-01-09

**Authors:** Alicia L Tsai, Davin Agustines

**Affiliations:** 1 Psychology, Claremont McKenna College, Claremont, USA; 2 Psychiatry, Olive View-University of California Los Angeles Medical Center, Los Angeles, USA

**Keywords:** oculocutaneous albinism, schizophrenia, genetic linkage, tyrosinase, albinism, melatonin

## Abstract

Oculocutaneous albinism (OCA) is an extremely rare skin disorder which occurs in 0.005% of the world population, whereas schizophrenia is a rare mental illness which affects 1% of the world population. Researchers have spent much time searching for the causes of schizophrenia, as they are still largely unknown. It was previously hypothesized that schizophrenia could be caused by a defect in melatonin metabolism, leading to increased melanin production and the production of hallucinogenic agent. However, this implies that albinos would be protected against schizophrenia (since they have little to no melanin production), and although rare, there have been several case reports of albinos with schizophrenia, refuting this hypothesis. Following their discovery of schizophrenic albinos, several researchers have instead wondered whether schizophrenia and albinism could actually be genetically linked. To further this discussion, we present a case report of a 25-year-old African-American male with OCA2 and schizophrenia. He was hospitalized after his mother discovered the existence of a BB that was lodged in his forehead from a failed suicide attempt in response to command auditory hallucinations. The BB was removed during his hospitalization, and he was psychiatrically stabilized on a combination of risperidone, lithium, and escitalopram.

## Introduction

Oculocutaneous albinism (OCA) is an autosomal recessive disorder defined by little to no melanin production in the eyes, skin, and hair, and is extremely rare, affecting 0.005% of the world’s population [1]. There are four main types of OCA (OCA1-OCA4), which primarily differ by the amount of melanin produced and so can be phenotypically distinguished by the amount of pigmentation in the eyes, skin, and hair of an individual (ranging from white/extremely pale to red). As a result of this decreased pigmentation, those with OCA can be subject to severe sunburns if not careful, and have a much higher susceptibility to skin cancer. Visual defects such as nystagmus and strabismus are often seen in those with OCA as well due to pigmentation deficits in the eyes [1].

Schizophrenia is a mental illness affecting approximately 1% of the world's population, and is characterized by behavior such as disorganized thinking and speech, delusions, hallucinations, and unusual body movements [2]. Previously, while searching for a cause for schizophrenia, Greiner and Nicholson (1965) noted that those with schizophrenia had increased levels of melanogenesis, and thus proposed the following hypothesis: in schizophrenic patients, the synthesis of melatonin from serotonin is defective and as a result, there is production of a hallucinogenic agent and an increase in melanin production. It was thought that the absence of 5-hydroxy-o-methyl transferase, which is necessary to form melatonin, could be the reason for such a defect in melatonin metabolism [3]. If true, this hypothesis would imply that those with decreased or no melanin production, such as albinos, should be protected from schizophrenia. Several researchers have searched for schizophrenic albinos in response to this hypothesis, and although extremely rare, a few cases of such co-existence have been reported [4-6]. These case reports not only refute the hypothesis that the amount of melanin is related to schizophrenia, as albinos can be schizophrenic, but some of these authors have even proposed that schizophrenia and albinism could actually be genetically linked. Such is the speculation in case reports by Barron (1976) and Clarke and Buckley (1989), who each separately found instances of multiple individuals with schizophrenia within families [7,8]. Whether OCA and schizophrenia are genetically linked is still undetermined, likely due to the rarity of both conditions occurring simultaneously and the paucity of literature regarding this topic-the last identified case report of a patient with both albinism and schizophrenia published on PubMed is from 1989 [4,8]. We hereby present a case of schizophrenia in a young albino male and will further the discussion exploring the fate of the original Greiner and Nicholson hypothesis and how modern techniques could point to a possible linkage of albinism and a certain subtype of schizophrenia.

## Case presentation

We report the case of a 25-year-old African-American male with OCA2, who was admitted in response to a phone call his mother made to emergency services after making threats to kill himself. The patient was admitted with symptoms of auditory hallucinations, paranoid delusions of people stalking him and stealing his thoughts, self-neglect, depression, and suicidal thoughts, which, according to his mother had increased when he became medication noncompliant. He had been hospitalized and discharged one month prior at a different facility, when he had shot himself in the forehead with a BB gun in response to command auditory hallucinations. His mother called 911 when she discovered two BBs lodged in his forehead. He had been released after a two-week stay, but continued to experience command auditory hallucinations to kill himself. His mother again called 911 when he expressed his urges to attempt suicide again. Physical examination revealed the presence of one BB in his forehead, as well as nystagmus and hypopigmentation over his entire body. Social history revealed no smoking or alcohol usage, but a urine test was positive for cannabis. An inquiry into family history was made, and it was noted that the patient has a paternal female cousin with albinism (with no current signs of schizophrenia) and maternal grandmother with schizophrenia. As for his immediate family, there were no other cases of either schizophrenia or albinism among his biological parents or three siblings. According to his mother, he had previously lived a rather normal life (although more on the withdrawn side) and schizophrenic symptoms had only developed a few years prior, when he was around 22 years old.

The patient was started on risperidone, which was uptitrated to 6 mg daily, and escitalopram at 10 mg daily for ongoing depressed mood. This medication combination lessened the intensity of auditory hallucinations significantly, but he remained very depressed and isolative on the inpatient psychiatric ward, and continued to express thoughts of hopelessness. Lithium augmentation was started, and lithium was uptitrated to 900 mg daily. With the addition of lithium, the patient’s neurovegetative symptoms of depression and his sense of hopelessness lessened. He ceased to have thoughts of suicide, and began to interact with treatment staff, as well as psychotherapeutic rehabilitation activities offered on the ward. He also allowed treatment team to consult with ENT (ear, nose, and throat) services to have the BB removed from his forehead. He agreed to engage with intensive wrap-around mental health services, along with ongoing psychosocial rehabilitative treatment offered as part of outpatient mental health treatment. He returned home with his mother after his positive, cognitive, and negative symptoms of schizophrenia lessened to a degree that he no longer expressed any hopelessness or wishes to die.

## Discussion

As the cause for schizophrenia is still not entirely known, researchers over the years have searched far and low attempting to piece together the basis for schizophrenia. A prior hypothesis by Greiner and Nicholson proposed that schizophrenia could be caused by a defect in melatonin metabolism which, as a result, would lead to hypermelanosis and the production of hallucinogenic agent [3]. This proposal implies that albinos would be protected from schizophrenia, as albinos have little to no melanin; however, it is now known that albinism and schizophrenia can occur together, with several identified case reports [4-6]. What is not known is whether this occurrence is due to chance, or whether there may be a clinically significant relationship and genetic linkage at hand-several researchers have speculated whether this could be the case after searching for and successfully finding schizophrenic albinos in response to the schizophrenia-melanosis hypothesis [7,8]. A previous pedigree study by Baron found that across two generations of a family, five members were albino and all five displayed symptoms of schizophrenia, leading Baron to believe that this showed very plausible indication of linkage [7]. There was another case report where two out of three siblings in a family had both schizophrenia and albinism, again raising the question of familial association and genetic linkage [8]. Our particular patient had a cousin who also had albinism. If our patient’s cousin were to develop schizophrenic symptoms in the future, having another familial association that pointed at potential linkage could be significant, as it would be hard to contribute such occurrence within a family to chance when these two traits almost never occur together. 

As genetics is now thought to be the main cause of schizophrenia, recent studies have focused on searching for the exact genes that lead to schizophrenia. There appear to be multiple genetic mutations across several genes that may play a role in the development of schizophrenia. More recent studies have found copy number variants in multiple genes being a factor in the development of schizophrenia, with mutations on chromosome 15 recently reported as being a schizophrenia risk factor [9]. Genes in the chromosome 11 breakpoint region were also previously examined, and upon further investigation, four candidate genes were identified as promising based on probable functional role and breakpoint proximity-one of these four genes was tyrosinase [10]. Another study also found tyrosinase to be a possible candidate for a predisposition to schizophrenia, since it could be involved in catecholamine metabolism [11]. As the classic form of OCA is caused by the lack of tyrosinase activity in the melanosome, it appears that there may be reason to suspect genetic linkage between schizophrenia and albinism [1,10]. Based on the current state of schizophrenia genetic research, as well as the established genetic causation of albinism, this question is certainly raised by our case report. As another point to consider, if schizophrenia and albinism were not genetically linked, there should be around 165 cases of schizophrenic albinos in the United States; if much more than 165 schizophrenic albinos have been identified, this could be additional indication of genetic linkage. 

Previous studies have shown that albinos already rank lower in psychological health [12,13]. It is therefore important that physicians who treat albino patients should consider the potential mental health issues that may also be affecting their patients, especially if future studies show that there is indeed a link between albinism and a potential cause of schizophrenia.

## Conclusions

Based on our case report and previous case reports in the literature we have reviewed, we can reject the schizophrenia-melatonin hypothesis, as it has been shown on multiple occasions that albinos can be schizophrenic. While we cannot yet be certain if there is a genetic linkage between the two traits, it is important to keep in mind that schizophrenia is multifactorial and even if a relationship is found, it likely would only explain a small number of cases and only a certain subtype of schizophrenia. However, it is still good to be aware of all the genetic issues that can contribute to cognitive issues even if they may be uncommon. 

## References

[1] Spritz RA (1994). Molecular genetics of oculocutaneous albinism. Hum Mol Genet.

[2] Escudero I, Johnstone M (2014). Genetics of schizophrenia. Curr Psychiatry Rep.

[3] Greiner AC, Nicolson GA (1965). Schizophrenia-melanosis. Lancet.

[4] Jurjus G, Moh P, Levy AB (1989). Oculocutaneous albinism and schizophrenia-like psychosis. J Nerv Ment Dis.

[5] Leibowitz MR, Dogliotti M, Hart G (1978). Schizophrenia and albinism. Dermatology.

[6] Pollack MH, Manschreck TC (1986). Oculocutaneous albinism and schizophrenia. Biol Psychiatry.

[7] Baron M (1976). Albinism and schizophreniform psychosis: a pedigree study. Am J Psychiatry.

[8] Clarke DJ, Buckley ME (1989). Familial association of albinism and schizophrenia. Br J Psychiatry.

[9] Kotlar AV, Mercer KB, Zwick ME, Mulle JG (2015). New discoveries in schizophrenia genetics reveal neurobiological pathways: a review of recent findings. Eur J Med Genet.

[10] Semple CA, Devon RS, Le Hellard S, Porteous DJ (2001). Identification of genes from a schizophrenia-linked translocation breakpoint region. Genomics.

[11] Petit J, Boisseau P, Taine L, Gauthier B, Arveiler B (1999). A YAC contig encompassing the 11q14.3 breakpoint of a translocation associated with schizophrenia, and including the tyrosinase gene. Mamm Genome.

[12] Attama CM, Uwakwe R, Onyeama GM, Igwe MN (2015). Psychiatric morbidity among subjects with leprosy and albinism in South East Nigeria: a comparative study. Ann Med Health Sci Res.

[13] Ezeilo BN (1989). Psychological aspects of albinism: an exploratory study with Nigerian (Igbo) albino subjects. Soc Sci Med.

